# Effect of cotton dust on lungs among female workers in cotton industry in northern Gujarat, India

**DOI:** 10.6026/97320630018255

**Published:** 2022-03-31

**Authors:** Gnanadesigan Ekambaram, Alkesh Vara, Shah Mansi Nileshkumar, N Sivasubramanian

**Affiliations:** 1 Department of Physiology, Nootan Medical College & Research Centre, Sankalchand Patel University, Visnagar, Gujarat, India; 2 IIIrd Year MBBS Student, Nootan Medical College & Research Centre, Sankalchand Patel University, Visnagar, Gujarat, India; 3 Department of Psychiatric Nursing, Nootan College of Nursing, Sankalchand Patel University, Visnagar, Gujarat, India

**Keywords:** Cotton dust, cotton industry workers, pulmonary function tests

## Abstract

Byssinosis is a disabling occupational lung disease caused by cotton dust. It is a well-known occupational respiratory disease in cotton industry workers caused by cotton dust pollution. Many studies have been documented the effects of cotton dust on pulmonary function among workers employed in cotton-spinning mills. However, little data exist on the prevalence of this disorder in female workers particularly in western part of India. The present study was conducted to analyze the effects of exposure to cotton dust on pulmonary functions among female workers. The study was designed to assess the effects of exposure to cotton dust on lung functions among female cotton industry workers. Study group comprises 50 Female workers of cotton industry and control group comprises 50 age matched females who were neither worked in cotton industry nor exposed to cotton dust. Information was collected using standardized questionnaires, physical examination and spirometric measurements. Student's T test was used to find the difference between spirometric parameters. All the respiratory parameters (FVC, FEV1, FEV1/FVC ratio, FEF 25-75 % PEFR and MVV) were reduced in cotton industry workers as compared with control subjects (p<0.0001) and no significant difference of SpO2 between groups. Cotton dust exposure makes huge impact on respiratory parameters of the cotton industry workers. This deterioration in respiratory health deteriorates with increasing duration of exposure. The health hazards caused by cotton dust should be controlled by creating awareness among the workers & employers.

## Background:

Byssinosis is a crippling occupational lung disease caused by cotton dust. It is a well known occupational respiratory disease among cotton mill workers induced by cotton dust pollution. Byssinosis also called Brown Lung or Brown Lung disease is a type of Pneumoconiosis caused by dust from cotton fibers. Inhaled dust stimulates histamine release which causes constriction of the air passages, making breathing difficult over time. Inhalation of the dust particles depends on its aerodynamic diameter, surrounding air's velocity, and breathing rate of the person. The inhaled fine cotton dust particles would get sedimented in the gas-exchange part of the lung tissue, where air flow is slow. These fine particles get lodged in the respiratory bronchioles within the central part of the acinus. The duration of exposure to cotton dust, type of dust, concentration of dust and the size of dust particle influence the effect on lungs [1]. The prevalence of respiratory problems related to cotton industry have been reported and varies from 2% in the USA in the late 1970s, 4 to 63% in England in the late 1950s [2] when compare with developed countries, prevalence rate is continues to be high in developing countries. The dust accumulates in the lung tissue and producing a distinct color that gives the disease its common name. Various epidemiologic studies of cotton industry workers have demonstrated evidence of lung involvement resulting from occupational exposure to cotton dust [3]. Initially workers have shortness of breath and tightness of chest on the first day of work. Hence it is also known as "Monday dyspnoea". Spirometry is crucial for evaluation of lung function both in clinical and occupational medicine and it is indicated at all the levels of prevention and its uses range from pre-employment evaluation or surveillance programs to clinical assessment of symptomatic subjects [4]. Recently pulmonary function tests (PFT) have opened a new beginning towards scientific approach in diagnosis, prognosis and management of lung abnormalities. The normal value ranges for pulmonary function tests (PFT's) will be adjusted for the subject's sex, height, and race [5 - check with author]. PFTs provide an objective and quantifiable measure of lung functions [6]. It permits an accurate and reproducible data of the working state of the lung tissue and allows assessment of severity level of lung pathology. The health imperilment of the women working in the cotton textile industry is much higher compared to male in other sectors. Most of the workers in these sectors are unfamiliar of this negative impact on their health and they mostly do not take any safety precautions at all times. A mortality study conducted in 1985 of women aged between 15 to 74 years found a raised proportional mortality ratio (PMR) from all causes of respiratory disease, including byssinosis, in cotton textile workers particularly among laborers. The Occupational Health Decennial Supplement published in 1995 reported a marked PMR for byssinosis than other diseases like chronic bronchitis and emphysema in female textile workers. Many research articles have been reported the negative impact of cotton dust on pulmonary function among workers employed in cotton-spinning mills. However, scarcity exists on the prevalence of this lung disorder in female workers particularly in western part of India. Working women have multiple roles in family and working sector as employee. Being subject to dual demands of home and workplace, they are liable to face crisis of adjustment which may cause physical and mental stress and strain. Many studies have been done to evaluate both acute and chronic exposure of cotton dust. Most of the epidemiological & experimental studies reported have focused on acute changes. The relationship between these and the chronic exposure particularly on female workers has not been clarified. This is necessary for proper protection and identification of people at risk. So far in India only limited numbers of studies have been conducted among female workers to highlight the occupational hazards and there is no recent study, which portrays clearly the effects of cotton dust exposure on lung functions among female workers in western part of India. The present study was conducted to analyze the effects of exposure to cotton dust on pulmonary functions among female workers. The study was determined to assess the effect of exposure to cotton dust on lung functions among female workers of cotton industry.

## Methodology: 

The present study was conducted in department of physiology, Nootan Medical College & research Centre, Sankalchand Patel University, Visnagar, Mehsana district. This study composed of two groups which contains totally 100 subjects. Group A (Study group) comprises 50 Female workers of cotton industry. The workers were selected from different cotton textile industries of Mehsana district. Group B (control group) comprises 50 age matched females who were neither worked in cotton industry nor exposed to cotton dust. Female subjects of cotton industry, relatives of patients attending various outpatient departments in the age group of 18 - 40 years, who come forward for the study voluntarily and satisfied the inclusion criteria, were recruited. Subjects on medication for Cardiovascular respiratory illness and Central nervous system (CNS) disorders, subjects with past and present history of CVS disorders, diabetes, psychiatric illness, subjects with history of drug abusing, smoking were excluded.

## Study procedure:

A elaborate clinical history of these volunteers were taken such as relevant past history, family history, drug history, personal history like alcoholism, smoking etc. A structured questionnaire was given to all volunteers (subjects) to obtain the details of occupational history and history of past or present respiratory illnesses. The present study was approved by the Institutional ethics committee (Approval no: IEC/NMCRC/APPROVAL/22/2021) and an informed consent was taken from all the subjects after explaining the test procedures and the goal of the study in local language. The anthropometric measurements such as height, weight, Body mass index (BMI), body surface area (BSA), lean body mass (LBM), Fat % were recorded for each subject by using standard procedure [7 - check with author]. SpO2 (Oxygen saturation) was measured with a digital handheld pulse oximeter (BPL smart oxy fingertip pulse oximeter, India) to investigate oxygen saturation (blood oxygen level) in blood. Participants rested at least 15 minutes before examination of SpO2. PFT was done with the help of spirometer (RMS Helios 401). The following variables were evaluated for spirometry: FVC (L) (Forced vital capacity), PEF (L/S) (Peak expiratory flow), FEF 25-75% (Forced expiratory flow), FEV1 (L) (Forced expiratory volume in first second of FVC), MVV (Maximum voluntary ventilation), and FEVI/FVC (%). The subjects were briefed about the procedure of conduction of spirometry and demonstration was given before initiation of procedure. The subjects were instructed to take deep inspiration from outside and then to expire as forcefully and as fast as she can inside the mouthpiece and recorded the activities. Using all aseptic techniques, the pulmonary function tests were conducted on subjects in standing position and wearing nose clips. Each test was repeated three times and the best results among the three were considered for analysis. Experiments were done in accordance with revised Helsinki Declaration of 2000.

## Statistical analysis:

Statistical analysis was performed with SPSS (Statistical Package for Social Sciences) (version 17.0). Results are expressed as mean ± standard deviation (SD) and assessed using Student's t-test. P-value < 0.05 was considered statistically significant.

## Results:

Table 1(see PDF) describes baseline (anthropometric) characteristics of the subjects. It can be seen that majority of the subjects were adults and all the subjects were females. There was no significant difference between the two groups (control & study group) in terms of anthropometric measures. Table 2(see PDF) describes the lung function parameters (Spirometry) of study and control group subjects. All the respiratory parameters (FVC, FEV1, FEV1/FVC ratio, FEF 25-75%, PEFR and MVV) were reduced significantly in cotton industry workers as compared with control subjects (Group B) (p<0.0001). The mean predicted values of FEV1 and FVC parameters were reported in Table 1(see PDF). Predicted value of FEV1 in study subjects were decreased significantly (p<0.0466), predicted FVC value was also decreased in study group however it was not statistically different (p<0.0802). The prevalence of various respiratory symptoms was shown in Table 2(see PDF) and the most common finding was reported is dyspnea followed by chest tightness & cough (16% & 20% respectively). No such symptoms were noted in control group (Group B). Figure 3(see PDF) shows Mean value of SpO2 (Oxygen saturation) in study and control groups. No significant difference of SpO2 between study and control groups (p<0.6612). Table 3(see PDF) shows duration of Cotton dust exposure among cotton industry workers. In the present study majority of the subjects had an exposure of at least 10 years or more showing an increased risk of developing lung diseases related to byssinosis.

## Discussion:

Women working in the cotton industry are more susceptible to various health problems much to their counter parts in other sectors. In the present study the anthropometric parameters of study and control group showed no significant difference thus making group comparable. In the present study, Forced vital capacity (FVC) was significantly decreased in cotton industry workers. This is supported by a report of Fishwick and Jadhav A.J et al [8,9]. Studies showed that cotton textile industry employees exposed to cotton dust reported acute reduction in FEV1 value due to hypersensitive airways. FEV1 value is reduced in both obstructive and restrictive disease. In the present study FEV1 is significantly decreased in cotton industry workers. More similar findings were reported in studies done by Wang XR et al [10], Dangi BM & Bhise AR [11]. Yonas Derso et al [12] studied lung functions on 83 healthy control subjects and 83 cotton-ginning workers and found significant decline in FVC and FEV1 due to prolonged exposure to cotton dust. FEV1/FVC ratio is an important obstructive type of lung illness in people who exposed to cotton dust. Byssinosis can give rise to both obstructive type and constrictive type of respiratory illness but usually predominant pattern is obstructive type of lung disease. FEV1/FVC value is normal in restrictive pattern but decreased in obstructive pattern. The present study shows decreased FEV1/FVC ratio markedly which is highly significant. This finding is matched with few studies [13, 14]. Jadhav A. J [9] studied lung functions in female workers and found significantly decreased in FEV1, FEV1/FVC ratio. It is in agreement with our report. In the present study we also found that cotton industry workers had less predicted FEV1 value compared to control group. Results of our study indicate impaired airway function. It is in agreement with Ioannis D. Anyfantis et al [15]. Peak expiratory flow rate (PEFR) is a reliable indicator of ventilation adequacy as well as airflow obstruction. In the present study, PEFR shows significant decrease in cotton industry workers. This suggests that cotton dust has an effect on PEFR. Similar results were obtained by Tiwari et al [16]. FEF 25-75 % is a more sensitive indicator of functional activity in middle/lower respiratory airways, but it is not a reproducible parameter as like FEV1. FEF 25-75 % is normal in restrictive pattern of lung disease. In the present study, FEF 25-75 % was significantly decreased in cotton industry workers. This is supported by Anitha et al [17 - check with author]. The maximal voluntary ventilation (MVV) is the largest volume of gas that can be transported into and out of the lungs in one minute by voluntary effort. The MVV depends upon the compliance of the thoracic cage and lungs and force of muscle. Maximal voluntary ventilation (MVV) is enormously decreased in patients with airway obstruction. In the present study mean MVV level was significantly decreased in cotton industry workers. Decrease in the value of MVV indicates increase in airway resistance. It is supported by X-R Wang et al [18], who studied effects of chronic exposure to cotton dust. Blood oxygen level is the amount of circulation of oxygen in the blood. A person's blood oxygen level is a good indication that shows how perfectly the body distributes oxygen from the lung tissue to the cells, which is important for maintenance of normal health. People with either organic dust exposure or other respiratory illness are necessary to keep an eye on their blood oxygen level. Low blood oxygen level (<95%) is associated with cardio-respiratory illness. Our study did not show clear association between oxygen saturation (SpO2) level between control and cotton industry workers. It needs further detailed analysis to understand oxygen saturation level in byssinosis. In the Present study the cotton dust exposed workers had more respiratory symptoms particularly dyspnea. The prevalence of respiratory symptoms in the current study was comparable to the findings of Nafees et al [19], who reported that 7.5% and 12.9% of cotton workers had chronic cough and chronic phlegm, respectively. We also reported cough, chest tightness and dyspnoea in cotton industry workers. These results were suggestive of impaired airway function and might be related to occupational exposure to cotton dust. We also found that workers in cotton industry had a higher risk of developing obstructive pulmonary diseases.

## Conclusion:

The Present study showed a strong coalition between cotton dust exposure in the cotton industry environment and lung function abnormalities. The pulmonary parameters are decreased significantly in the subjects working in cotton industry. This suggests that more aggressive measures must be taken in the workplace and enforcing the use of personal protective equipment, such as respirators and face masks. The health hazards caused by cotton dust should be controlled by creating awareness such as regular health check-ups, personal protective measures and proper ventilation among the employees & employers and thereby ensuring the quality of life of the cotton textile industry workers.
